# *FlɛX*: a computer vision program to evaluate strain in flexible crystals

**DOI:** 10.1107/S1600576723008282

**Published:** 2024-03-15

**Authors:** Benjamin Hsieh, Lai-Chin Wu, Arnaud Grosjean

**Affiliations:** a National Synchrotron Radiation Research Center, Hsinchu 30076, Taiwan; HPSTAR and Harbin Institute of Technology, People’s Republic of China

**Keywords:** flexible crystals, computer vision, Euler–Bernoulli beam theory

## Abstract

The program *FlɛX* (flexural ɛ for xtals) has been developed for a quick, easy and accurate evaluation of the maximum deformation reached in flexible crystals from a simple optical microscope picture. The program takes advantage of computer vision libraries to find the contours of a bent crystal and fit these to semicircles. It can then calculate the theoretical maximum deformation along its long axis using equations from the Euler–Bernoulli beam theory.

## Abstract 2

A program has been developed to assist researchers interested in bending crystals to evaluate crystal deformation from a single optical image.

## 1. Introduction

Despite crystals being often perceived as hard and brittle, numerous examples exhibit appreciable deformation (Ahmed *et al.*, 2018 ▸). The mechanical properties of these crystals are intriguing, and so developing methods to measure and quantify their deformation can prove helpful in studying such properties.

Though there is growing interest in the mechanical properties of flexible molecular crystals, these properties under bending are almost never evaluated. Indeed, it is extremely challenging to obtain samples of this type of materials that are sufficient in size for mechanical testing. By applying image-processing techniques, it is possible to, at least in part, fill the gap arising from the lack of mechanical testing. Image-processing techniques have already been successfully applied for macroscale samples (Kromanis & Kripakaran, 2021 ▸; Carmo *et al.*, 2019 ▸, 2013 ▸) and can be adapted for smaller sized samples. Specifically, image-processing techniques applied to macroscale samples rely on a preliminary marking of the sample, which is not possible for subcentimetric samples. Therefore, sample edge detection is required. The physical relationship can then be used to obtain useful mechanical information.

**Figure 1:**
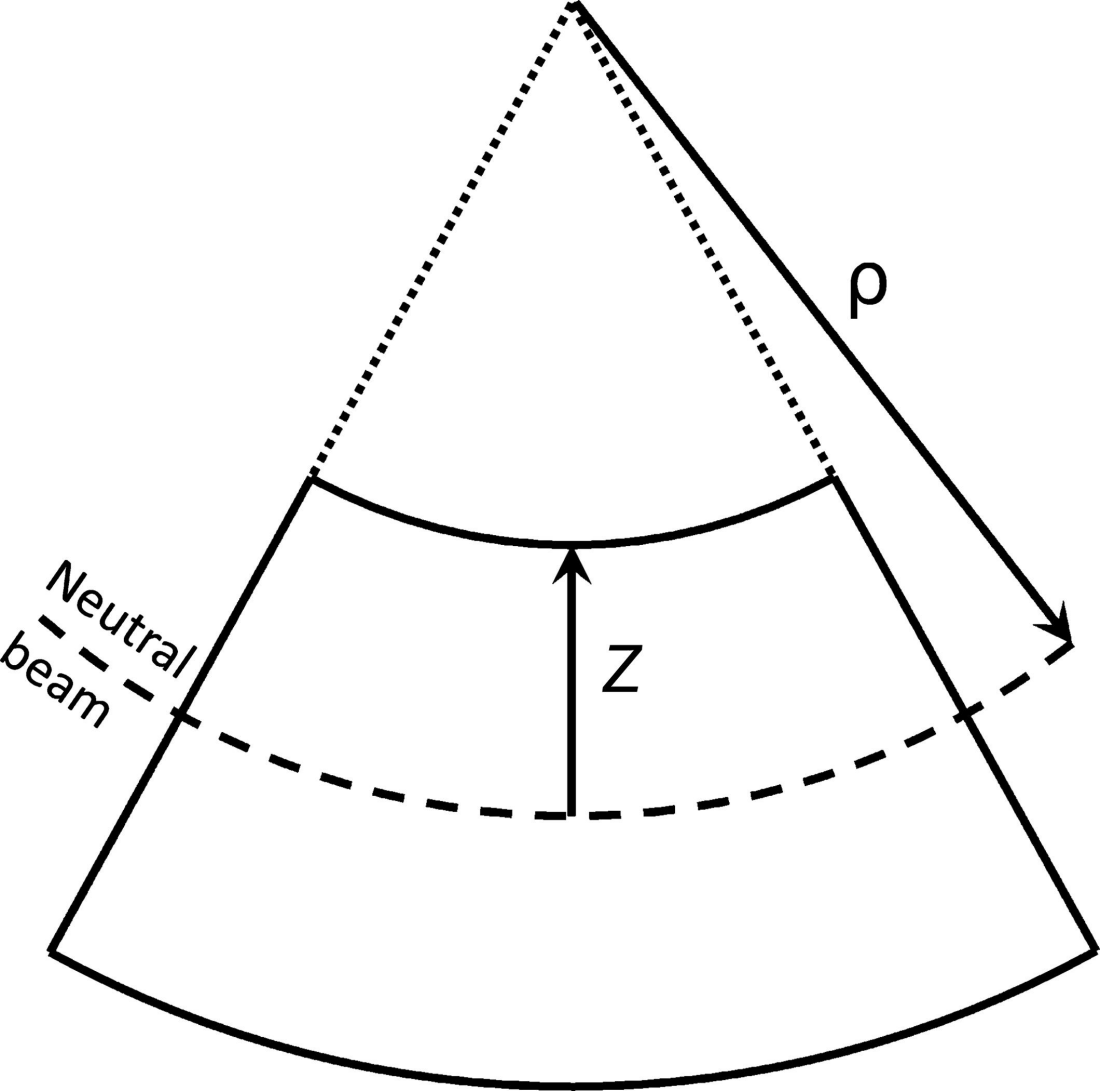
Schematic representation of a bent crystal with parameters *z* and ρ represented, with the equation for strain ɛ = *z*/ρ.

In perfect bending conditions, a beam or crystal deflects with the fibers on one side compressing and those on the other expanding, forming an arc. According to the Euler–Bernoulli beam theory (Gere & Goodno, 2013 ▸), the deformation (strain, ɛ) is given by *z*/ρ, where *z* is the distance from the neutral axis and ρ is the radius. With this equation, the theoretical maximum deformation of a crystal can be evaluated once its thickness and radius of curvature are known (Fig. 1 ▸).

While measuring the thickness and radius of curvature by hand could prove to be tedious and inaccurate, it is possible to take advantage of automation algorithms to make the process reasonably fast, easy and reliable. The purpose of the program *FlɛX* (flexural ɛ for xtals) is therefore to provide the maximum deformation of a crystal based on a simple optical microscope image.

## 2. Methods

The program *FlɛX* first receives an image file and then uses computer vision tools from *OpenCV* (Kaehler & Bradski, 2017 ▸) and *NumPy* (Harris *et al.*, 2020 ▸) to identify the crystal contour. The image can be blurred to reduce noise and improve the continuity of contour lines. A fit of the contour to semicircles using *SciPy* (Virtanen *et al.*, 2020 ▸) allows the program to obtain the radius and thickness of the crystal. Finally, *FlɛX* calculates the maximum deformation along the long axis using Euler–Bernoulli beam theory (Gere & Goodno, 2013 ▸) and displays the outputs with *Matplotlib* (Hunter, 2007 ▸). A refit without 10% of the points will also be carried out to remove any potential outliers. The errors are estimated using the principles of uncertainty propagation (Taylor, 1997 ▸). However, this estimation does not include the uncertainty from contour finding.

## 3. Test examples

Though the program is intended for the study of flexible crystals, it can, fundamentally, be used to evaluate the deformation of any bent beam. Therefore, it has been tested on a few common materials, such as a bent polymer (Nylon 6,6) beam (zip tie), a bent steel beam (metallic ruler) and a bent wooden (birch) beam (chopstick) (see the supporting information). The deformations obtained of 2.6836 (9)%, 0.1996 (9)% and 0.9134 (6)%, respectively, are in perfect agreement with the expected values for these three types of materials (Ward *et al.*, 2013 ▸; ASM, 2007 ▸; Forest Products Laboratory, 2010 ▸). The results also show the ability of the algorithm to deal with very small deformation cases (steel) and low-quality images (wood).

**Figure 2:**
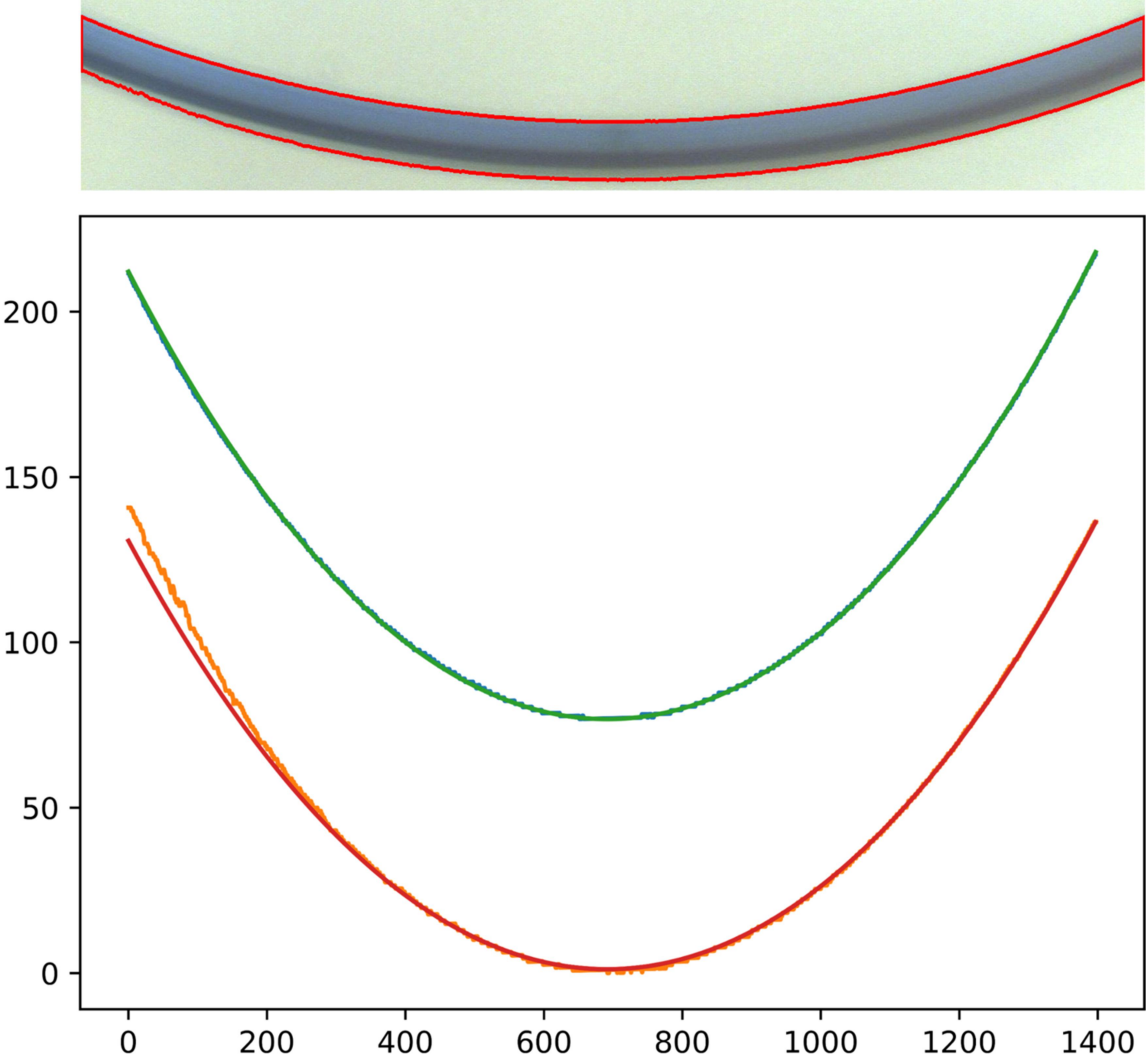
(*a*) Crystal contours automatically obtained. (*b*) Arc fitting of the contours to obtain the thickness and curvature of the crystal (pixels). The inside and outside contours are marked in blue and orange, respectively, and the green and red lines correspond to the inside and outside fitted arcs, respectively. (Here, the blue line is overlapped by the green line.)

The program has also been tested against a previously published image of a bent [Cu(acac)_2_] crystal (Worthy *et al.*, 2018 ▸). Fig. 2 ▸ shows the contours obtained from the optical image as well as the fitted arc. A small part of the outer arc was poorly fitted due to an optical reflection on the crystal, but the algorithm effectively discarded these data points. In this example, the crystal was not perfectly aligned perpendicular to the camera, leading to an overestimation of the thickness, so an adjustment was required. The degree of misalignment can be found using a modified version of the current algorithm and some photographs of the crystal from the side (Worthy *et al.*, 2018 ▸). Subsequently, a correction can be applied using simple trigonometry. Micro-focused X-ray diffraction (µ-XRD) mapping experiments on this crystal have shown that the deformation was 1.40 (3)% on the outside of the bend and 1.83 (3)% on the inside (Worthy *et al.*, 2018 ▸). This means that the neutral deformation beam on this crystal is actually not centered. Even though the reason for this displacement of the neutral axis is not apparent, an additional correction based on ratios of the deformations can still be applied to obtain final deformations corresponding to the inner and outer edges of the crystal. The final deformations obtained are 1.437 (1)% and 1.864 (1)% on the inner and outer edges of the crystal, respectively, closely matching the results from the µ-XRD mapping.

These examples demonstrate the effectiveness and accuracy of the program.

## 4. Program availability and requirements

The program is available via a web application at https://flexure-deformation-xtals.anvil.app/, which accepts input in standard image formats (*.png, *.jpeg *etc*.).

After extensive testing, it is clear that *FlɛX* can compute deformation as intended in most circumstances. However, some requirements exist for everyone interested in using this program. The crystal has to be in near-perfect bending conditions, *i.e.* it has to undergo four-point bending conditions instead of three-point bending. In addition, the crystal has to be a continuum medium, and there cannot be cracks along the middle section.

The crystal should be perfectly aligned perpendicular to the camera when an image is taken, otherwise the thickness or curvature will be overestimated depending on the case (as shown previously). The crystal should be the only object in the picture; no bending tools or equipment should be visible. Shadows and reflections should be avoided, and the image should have reasonable resolution and contrast.

Some notable limitations do exist for *FlɛX*. As shown in the example, *FlɛX* cannot determine if there is a displacement of the neutral axis. Furthermore, it cannot determine whether the deformation is plastic, elastic or both from an image only. A possible method to accurately differentiate plastic and elastic deformation would be to apply the same process to a video. If the crystal conditions before and after bending were available to the program, the percentage of the deformation that is elastic could be computed.

## 5. Conclusions

The program *FlɛX* has been developed to enhance the study of flexible crystals. First, the program finds the crystal contour with the computer vision library *OpenCV* (Kaehler & Bradski, 2017 ▸). Then, the deformation can be quickly and accurately determined using the Euler–Bernoulli beam theory (Gere & Goodno, 2013 ▸). This tool could prove useful to investigate flexible crystals more accurately. Further development could be made to distinguish elastic and plastic deformation. This type of algorithm could also be adapted to automate crystal centering on XRD instruments.

## Supplementary Material

Supporting figure and table. DOI: 10.1107/S1600576723008282/iu5042sup1.pdf
