# Supplementary material for: FlɛX: a computer vision program to evaluate strain in flexible crystals
Source: J Appl Crystallogr. 2024 Mar 15;57(Pt 2):552–4. doi: 10.1107/S1600576723008282 (PMC11001399; doi:10.1107/S1600576723008282)
Supplement: Supplementary file 1 [file j-57-00552-sup1.pdf]

JOURNAL OF APPLIED CRYSTALLOGRAPHY

Volume 56 (2023)

Supporting information for article:

FlεX: a computer vision program to evaluate strain in flexible crystals This article is part of IUCr2023.

Benjamin Hsieh, Lai-Chin Wu and Arnaud Grosjean

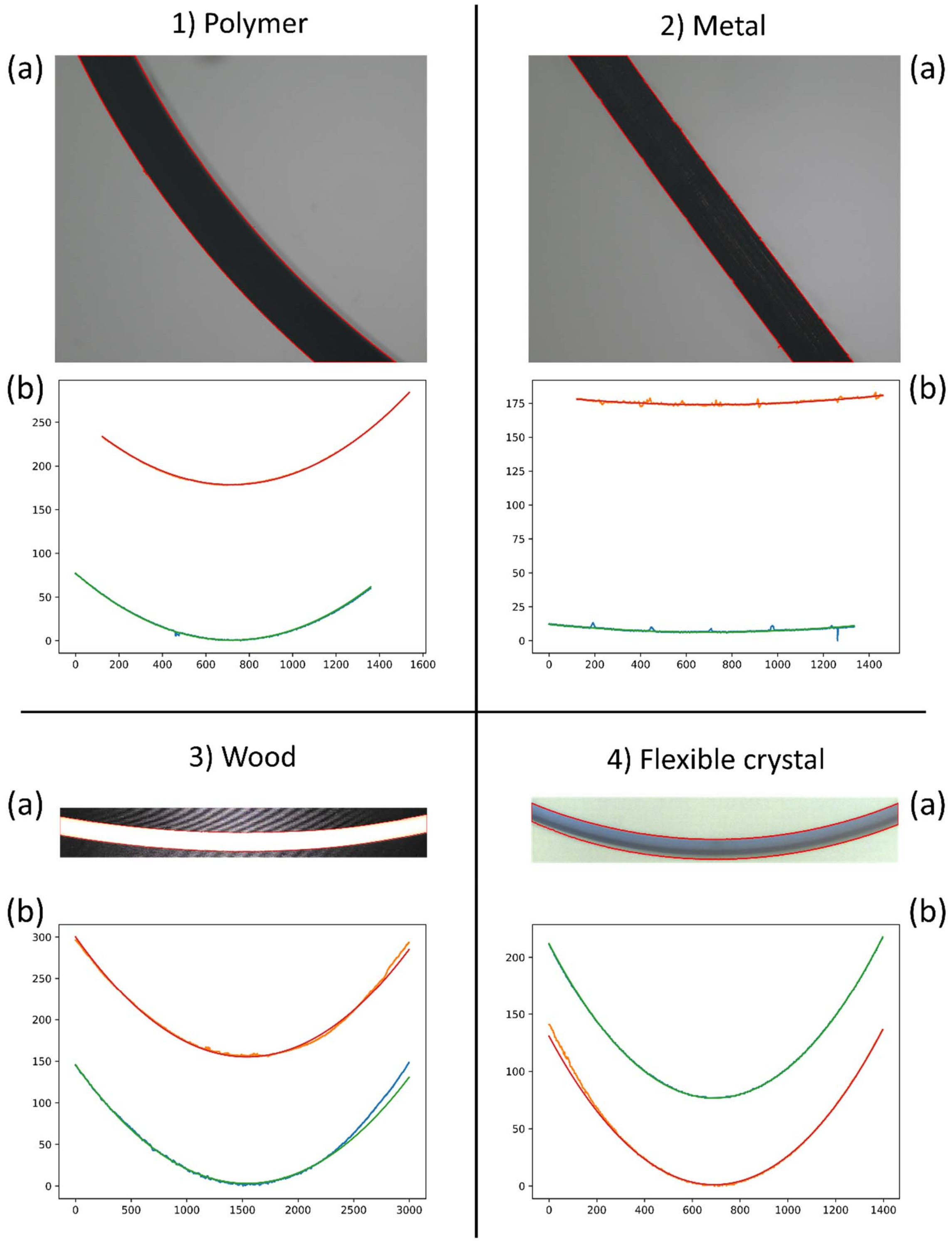

Figure S1 Crystal contours automatically obtained for test materials. 1) Polymer (Nylon 6,6), 2) Metal (Steel), 3) Wood (Birch) and 4) flexible crystal ([Cu(acac)$_2$]). a) obtained contours and b) fitted arcs.

Table S1 Results obtained by FlεX for the tested materials.

Material	Thickness (pixels)	Mid Radius (pixels)	Long Axis Max Deformation (%)
Polymer	177.93(2)	3315(1)	2.6836(9)
Metal	167.71(2)	42004(200)	0.1996(9)
Wood	152.02(5)	8322(6)	0.9134(6)
Flexible crystal (uncorrected)	75.61(2)	1871.9(5)	2.0195(7)
